# Metalenses phase characterization by multi-distance phase retrieval

**DOI:** 10.1038/s41377-024-01530-1

**Published:** 2024-08-06

**Authors:** Bowen Liu, Jialuo Cheng, Maoxiong Zhao, Jin Yao, Xiaoyuan Liu, Shaohu Chen, Lei Shi, Din Ping Tsai, Zihan Geng, Mu Ku Chen

**Affiliations:** 1 State Key Laboratory of Surface Physics, Key Laboratory of Micro- and Nano-Photonic Structures (Ministry of Education) and Department of Physics, Fudan University, 200433 Shanghai, China; 2 Department of Electrical Engineering, City University of Hong Kong, Kowloon, Hong Kong SAR China; 3 State Key Laboratory of Terahertz and Millimeter Waves, City University of Hong Kong, Kowloon, Hong Kong SAR China; 4 Institute for Nanoelectronic Devices and Quantum Computing, Fudan University, 200438 Shanghai, China; 5 Collaborative Innovation Center of Advanced Microstructures, Nanjing University, 210093 Nanjing, Jiangsu China; 6 Shanghai Research Center for Quantum Sciences, 201315 Shanghai, China; 7 Centre for Biosystems, Neuroscience, and Nanotechnology, City University of Hong Kong, Kowloon, Hong Kong SAR China; 8 Institute of Data and Information, Tsinghua Shenzhen International Graduate School, Tsinghua University, 518071 Shenzhen, Guangdong China

**Keywords:** Metamaterials, Imaging and sensing, Optical metrology

## Abstract

Metalens, characterized by their unique functions and distinctive physical properties, have gained significant attention for their potential applications. To further optimize the performance of metalens, it is necessary to characterize the phase modulation of the metalens. In this study, we present a multi-distance phase retrieval system based on optical field scanning and discuss its convergence and robustness. Our findings indicate that the system is capable of retrieving the phase distribution of the metalens as long as the measurement noise is low and the total length of the scanned light field is sufficiently long. This system enables the analysis of focal length and aberration by utilizing the computed phase distribution. We extend our investigation to measure the phase distribution of the metalens operating in the near-infrared (NIR) spectrum and identify the impact of defects in the sample on the phase. Additionally, we conduct a comparative analysis of the phase distribution of the metalens in air and ethanol and observe the variations in the phase modulation of the metalens in different working mediums. Our system provides a straightforward method for the phase characterization of metalens, aiding in optimizing the metalens design and functionality.

## Abstract (web summary)

We developed an intensity-based metalens phase retrieval method, revealing defects’ impact on phase and, for the first time, measuring the working medium’s influence on metalens.

## Graphical abstract

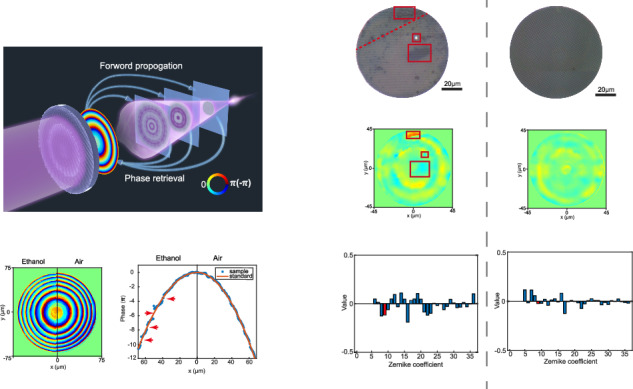

## Introduction

Metalens is a kind of optical metasurface composed of metaatoms for manipulating the amplitude, phase, and polarization of the incoming light^1–3^. Unlike traditional refractive lenses, metalens can modulate the wavefront from plane to spherical at an interface. It has garnered widespread attention due to its novel physical properties and promising potential applications. The significant advantages of metalenses are novel properties, lighter weight, high efficiency, compact size, and low-energy consumption. Metalenses have been demonstrated for beam shaping^4^, focusing^5,6^, imaging^7,8^, spectral imaging^9,10^, polarization generation and analysis^11,12^, second-harmonic generation^13^, color-routing^14^, light-field sensing^15–17^, and high-dimensional optical quantum source^18^. Currently, research on metalens predominantly focuses on their design, functionality, and fabrication, with relatively limited attention given to the aspect of characterization.

As a phase-modulated optical functional component, phase distributions are the key parameter of metalens. However, in the optical frequency band, it is difficult to measure the phase distribution directly. Instead of measuring phase, the mainstream measurement methods for characterizing metalens currently involve light field scanning and electron microscopy. While light field scanning can capture the functional information of metalens, it lacks the ability to provide localized optical field modulation details, which are crucial for further optimizing the metalens’ performance. Electron microscopy imaging methods can capture the topography details of the sample’s structure but are unable to provide insight into the optical response of the sample.

For a more comprehensive characterization of the metalens performance, precise measurement of the phase is essential. Existing methods for measuring the phase of metalens include quadriwave lateral shearing interferometry (QLSI)^19^ and off-axis interferometry^20^, both effective for measurements in the visible band. QLSI methods are achieved through the use of a 2D grating, which separates the incident wavefront into four distinct parts at different positions with different incident directions on the image plane and engages in mutual interference. The interference pattern yields information about the phase gradient of the test wavefront, and the phase distribution is deduced subsequently through path integration. This approach faces challenges in mitigating the impact of the grating itself and encounters lower energy utilization efficiency. The higher-order diffraction of the grating and the path integration process can introduce errors into the system. Off-axis interferometry involves introducing an off-axis reference beam, which interferes coherently with the sample beam, enabling measurement of the sample’s phase distribution. This method necessitates the establishment of a relatively complex optical setup coupled with stringent requirements for the coherence of the light source.

In this work, we develop a metalens phase-measuring system using multi-distance phase retrieval (MDPR)^21–23^. This method can calculate the phase distribution by measuring the light intensity distribution at various distances on the sample exit plane^24^. The convergence of the system itself and its sensitivity to different noises are discussed. Using this approach, we measured the phase distribution of a metalens working at near-infrared (NIR) and another metalens working at 405 nm in ethanol. The phase distribution allows for the calculation of wave aberration and other key parameters of the metalens.

## Result

### Phase retrieval from the multi-distance intensity distribution

**Fig. 1:**
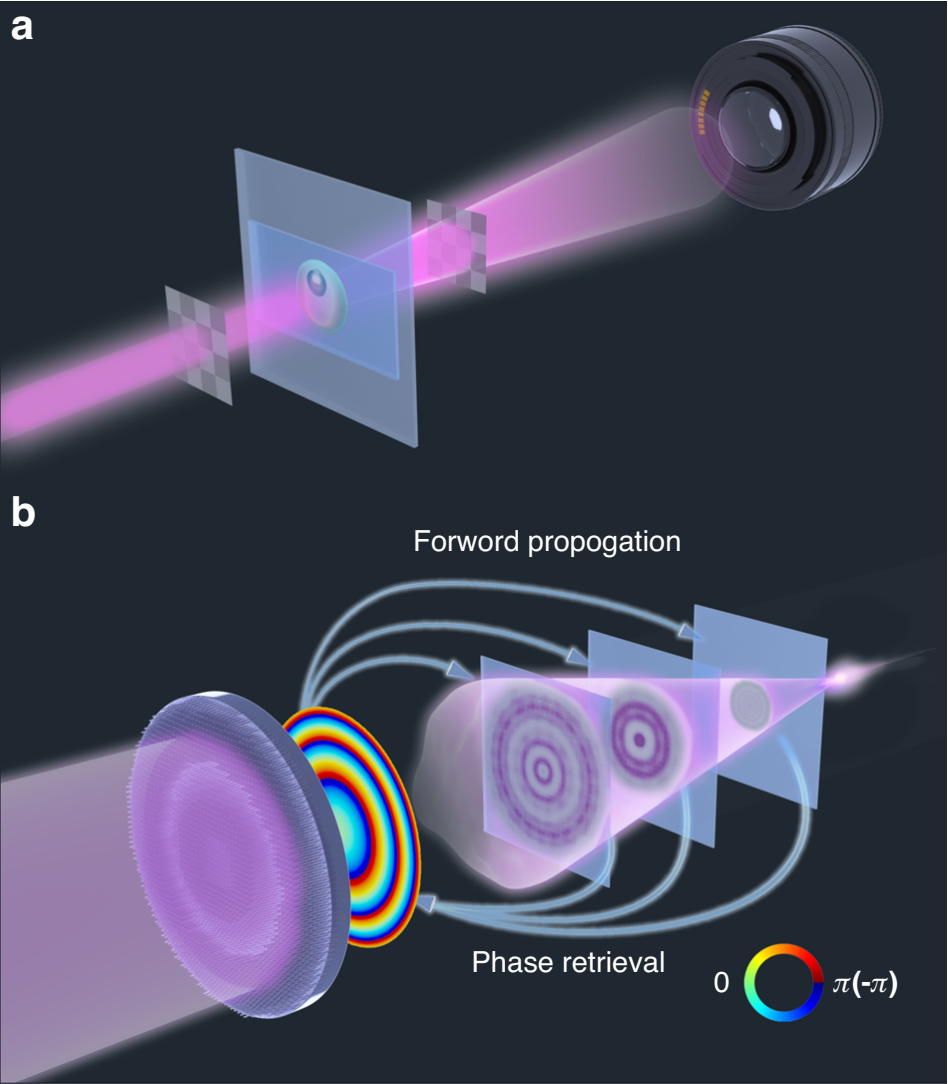
Illustration of phase retrieval. **a** Illumination encoding and measurement encoding. **b** Multi-distance phase retrieval

The fundamental problem in computational holography lies in determining the missing phase information from intensity measurement. In a linear optical system, the relationship between the directly measured intensity distribution $y\in {R}^{n}$ and the light field distribution to be determined $x\in {C}^{m}$ is given by

1 $$y={{\rm{|}}{Ax}{\rm{|}}}^{2}$$

where transfer matrix $A\in {C}^{n\times m}$, is determined by the measurement system. To enhance the acquired information *y* in measurements, it is necessary to expand the dimensions of matrix *A* by encoding the optical field. Following the encoding scheme, non-point-to-point computational holography can be divided into illumination encoding^25^ and measurement encoding^26^, as shown in Fig. 1a. A typical illumination encoding system is the Fourier ptychographic microscope (FPM)^27^, which achieves high-resolution holographic field imaging by altering the incident angle of illuminating light. This method finds favorable applications in imaging weakly scattering biological samples. However, traditional encoding methods may encounter issues when dealing with strongly scattering metalens samples. For illumination encoding systems, different encoding illuminations may introduce angular dispersion of the sample, resulting in different optical responses of the metalens measured under different illumination encodings. Using a spatial light modulator for encoding measurements may result in a part of light passing through the metalens being unreceived by the optical path.

In the MDPR system, collecting intensity distribution from different locations is also a form of encoding at the measurement terminal^28,29^, and transfer matrix *A* is determined by free-space angular spectrum diffraction

2 $${A}_{l}={F}^{-1}{D}_{l}F$$

where *F* represents the Fourier transform, *D*_*l*_ is the distance between the sample surface and the position of the *l*th intensity measurement.

The measurement process of the MDPR system is demonstrated in Fig. 1b. The sample is illuminated by a normal incident collimated laser beam, and several intensity distribution images of the focused light field are detected starting from the sample surface and scanning in the direction away from the sample at equal intervals. The measured intensity distributions are fed into the iterative algorithm, which iteratively refines a complex light field until it converges to a solution that accurately matches the measured intensity distribution.

### Phase retrieval algorithm for the MDPR system

**Fig. 2:**
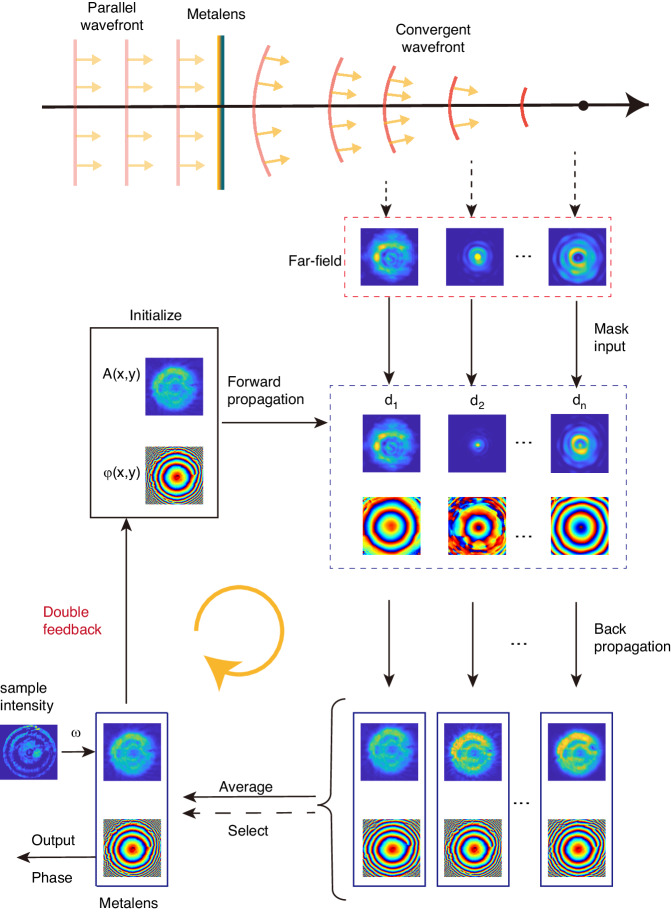
Algorithmic flowchart for phase retrieval from intensity images captured at different positions

Figure 2 shows the algorithmic flowchart of far-field amplitude-only speckle transfer (FAST)^30,31^, which retrieves the phase from intensity distribution through iteration. The key aspect of the phase retrieval algorithm is how to incorporate intensity distributions measured at different positions during the iterative process, ensuring the integration of information^32–34^. The core idea of our iterative algorithm is as follows: Initially, a random field distribution $A\left(x,y\right){{\rm {e}}}^{i{\rm{\varphi }}\left({\rm{x}},{\rm{y}}\right)}$ is intitialized at sample plane and propagated forward to a distance *d*_*i*_ from the sample surface using angular spectrum propagation. Retain its phase distribution and replace the calculated intensity distribution with the measured one. The updated far-field field distributions are then individually propagated backward to the sample plane, resulting in ***N*** corresponding sample plane field distributions. An average of the complex field distributions from these *N* sample plane field distributions is computed, resulting in a sample plane field distribution that integrates intensity information from *N* positions. Then, we utilize a method based on Kalman filtering to incorporate the measured sample surface intensity distribution *I*_0_ to update the sample surface light field. Subsequently, the far-field distribution at ***N*** positions is calculated using the averaged sample plane field distribution. The phase information of these ***N*** position field distributions is extracted, and selected regions of intensity are replaced with the measured intensity distribution, which is updated field distribution for the next iteration. By progressively introducing intensity distribution information during iterations, the algorithm converges toward the actual complex optical field distribution on the sample plane. The double feedback aspect enhances algorithm stability by incorporating information from the previous iteration. The “select” step in the algorithm aims to filter out images with significant measurement errors as the algorithm approaches the distribution of the measured light field, thereby enhancing the robustness of the algorithm. For details of the double-feedback process, mask function, kalman-filtering, and select step, refer to the Supplementary Information.

### Convergence and robustness of the MDPR system

**Fig. 3:**
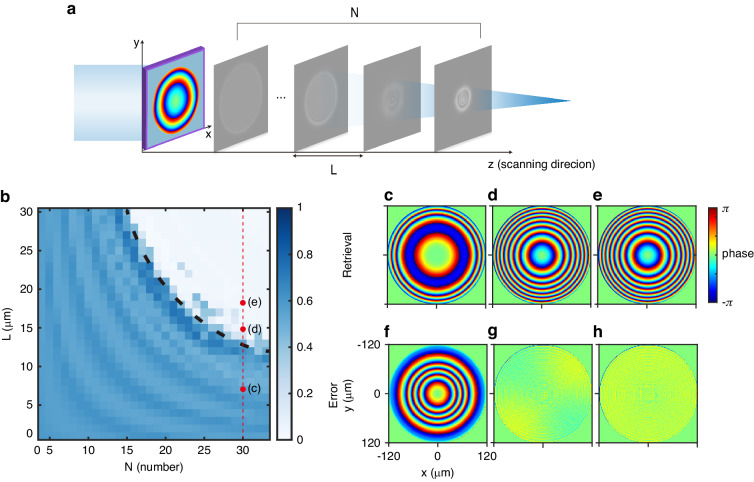
Convergence of MDPR system in metalens measurement with sampling picture number *N* and distance between pictures *L*. **a** The measurement setup of MDPR system for metalens. **b**–**h** Numerical simulation of a metalens working at 1550 nm with 120 μm in radius and 600 μm in focal length, (**b)** normalized MSE distribution with the number of sampling images and distance between them. Phase retrieval results for cases (**c**–**e**) and corresponding error analyses (**f**–**h**) based on different distances between sampling images. The retrievals were conducted with *N* = 30, and the parameter *L* varied: 7 μm for (**c**), 15 μm for (**d**), and 18 μm for (**e**)

For weakly scattering samples, phase retrieval from intensity distribution at different distances is an extremely ill-posed problem since the intensity distribution does not change significantly with distance. Metalens are strongly scattering samples, and parallel light will concentrate to a point after passing through the metalens, leading to rapid changes in intensity distribution. To ensure that MDPR for metalens can converge to the real field, it is necessary to ensure the number of measurements ***N*** and the difference between pictures is sufficient, where the latter is determined by the distance between sampled images ***L*** for a given metalens. Figure 3b demonstrates the relationship between the normalized mean square error (MSE) of retrieval results and the true values concerning the sample number *N* and the sampling distance *L*. It is worth noting that the phase calculated from intensity distribution may undergo an overall phase shift compared to the standard value. While this contributes to the mean square error (MSE), it does not affect the phase information of the metalens. For this reason, the difference between retrieval results and standard phase should be expanded as a Zernike function, and the contribution of phase shift and tilt should be excluded in MSE. There is a clear boundary in Fig. 3b, above which the algorithm can obtain the correct phase distribution. This boundary can be approximated as an inverse proportional curve *N* · *L* = 450 μm. In cases where the total scanning length exceeds 450 μm, the system can converge to the real value, indicating that the system has captured sufficient optical field variations. Figures 3c–h shows the results of the MDPR system as the sampling distance rises. Insufficient sampling intervals result in failure to converge to the real value.

**Fig. 4:**
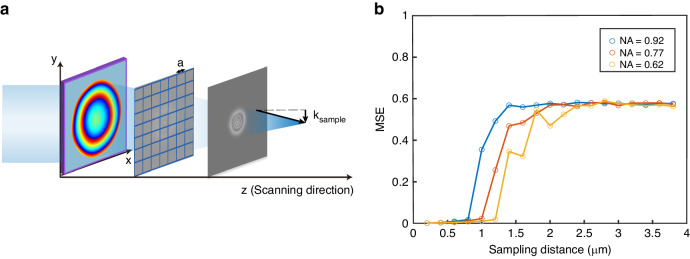
Convergence of MDPR system in metalens measurement with resolution changes. **a** Sampling the optical field intensity into discrete values with a given sampling intervals *a*. **b** MSE for retrieval results with different resolutions for metalens working at 1550 nm with different NA

The sampling resolution also affects the convergence of the MDPR system. The resolution of the measuring system is determined by the numerical aperture (NA) of the objective lens in the imaging optical path and pixel per inch (PPI) at the sample surface. When the NA of the sample exceeds that of the objective lens, light at large angles in the optical path will be filtered by the objective boundary. This will result in the phase information in the high-frequency region being unable to be received. If the sampling frequency of the measuring system is less than the spatial frequency of metalens, there will be a large difference between the real light field propagation behavior and the angular spectrum diffraction results shown in Eq. (2). Consequently, the retrieved phase distribution may significantly deviate from the real value. The trend of MSE between retrieval phase distribution and down-sampled standard phase distribution varies with the change of sampling interval *a*, as depicted in Fig. 4b. As the sampling interval increases, there is a turning point in the variation of the MSE. Before this point, the system can accurately retrieve the phase distribution with minimal error. The turning point determines the spatial bandwidth utilization (SBU) of the system, which is the ratio of sampling frequency $\frac{\pi }{a}$ and transverse wave vector of the focused optical field ${k}_{{{\rm {sample}}}}=\frac{2\pi }{\lambda }{{\rm {NA}}}$. For metalens with different NA, as shown in Fig. 4b, the SBU ranges from 0.95 to 0.99, indicating that the measurement system has effectively utilized the highest theoretical bandwidth. This provides the limitations of the measurement system on the sample. The sampling theorem requirements are given by

3 $$\frac{{\rm{\pi}}}{a} \,> \,\frac{2{\rm{\pi }}}{{\rm{\lambda }}}{{\rm {NA}}}$$

In which *a* represents the sampling interval, and NA represents the numerical aperture of the sample, *λ* is the wavelength. The field of view limitation is given by

4 $$N\cdot a\, >\, 2r$$

we can obtain the relationship between the number of samples, numerical aperture, and sample size. That is, the spatial bandwidth-product limitation of the sampling system

5 $$N > \frac{4r{\rm{NA }}}{{{\rm {\lambda}}}}$$

**Fig. 5:**
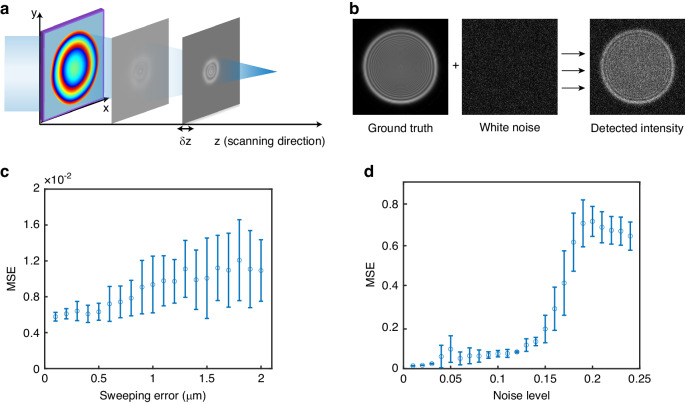
Phase measurement accuracy as a function of measurement error. **a** Phase retrieval in the presence of uncertainty in the *δz* of the translation stage position. **b** The measured results are superimposed with the CCD noise over the ideal intensity distribution. **c** MSE with the error of translation distance of displacement stage. **d** MSE with the error of intensity measurement. noise level is the ratio of intensity measurement noise level to intensity of incident uniform plane light

Errors may also be introduced during measurement. Experimental measurements are susceptible to errors primarily stemming from two sources: errors in the translation distance of the displacement stage and errors in intensity measurement. The way the measurement error is transferred to retrieval distribution is shown in Figs. 5a, and b. Note that due to the uncertainty of the impact of random noise, various noises are generated at the same magnitude to determine the MSE distribution. As the uncertainty of the displacement stage increases, the MSE rises in a nearly linear trend in Fig. 5c, while there is a jump for MSE when the intensity noise level rises in Fig. 5d, below which the effect of intensity noise is small.

For most micrometer-level displacement stages used in optical field scanning, achieving a unique uncertainty of 1 μm is easily achievable. The requirements for image white noise necessitate the careful selection of the camera’s integration time and incident light intensity to ensure that the noise level remains below the threshold at transition points.

### Experiment

Two metalens with identical designed parameters, working at 1560 nm, are measured. The design focal length is 220 μm, with a radius of 45 μm and a numerical aperture of about 0.2. The illumination is provided by a filtered supercontinuum laser with a bandwidth of 10 nm. Hence, its temporal coherence is insufficient to support interference measurements.

**Fig. 6:**
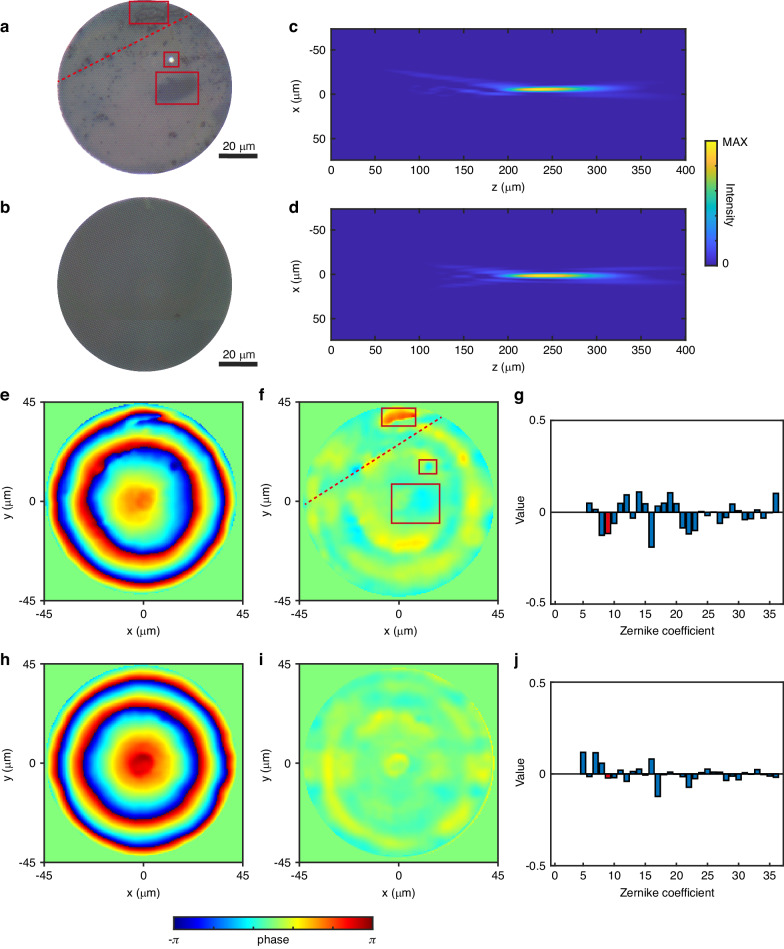
Phase measurement for metalens working at 1560 nm. **a**, **b** Optical microscope images depicting the sample with defects and the defect-free sample. **c**, **d** Experimentally measured optical field distributions for the sample with defects and the defect-free sample. Phase distribution, wave aberration, and Zernike expansion coefficients are illustrated for the sample with defects in (**e**–**g**) and the defect-free sample in (**h**–**j**)

Figures 6a, and b show the optical photographs of the two samples. One of the samples exhibits a series of defects on its surface. Figures 6c, and d show the focused intensity distribution of these two samples. The focusing efficiency of the sample with defects is 0.27, while that of the defect-free sample is 0.51, as determined from the intensity distribution. The presence of defects has resulted in a decrease in focusing efficiency.

The phase retrieval results for these two samples are presented in Figs. 6e, and h. In our scanning system, the uncertainty of the translation stage is 0.3 μm, with a lateral resolution of 578 nm. For the NIR camera, after subtracting dark noise, the intensity noise in the optical field measurement can be neglected (refer to Fig. S12). Starting from the sample surface, we selected 30 images with a spacing of 6 μm for phase retrieval, which is sufficient for the convergence of phase retrieval.

The phase distribution analysis for Figs. 6e, and h reveals an actual focal length of 250 μm for the sample with defects and 243 μm for the defects-free sample. It is worth noting that due to the edge diffraction effects of the sample, the focal length obtained from the phase differs from the position where the strongest point appears in the scan field. After subtracting the standard phase at the corresponding focal length, wave aberrations are calculated from the phase distribution as shown in Figs. 6f, and i. The wave aberration for the sample with defects is greater than that for the defect-free sample, with corresponding variances of 0.13 and 0.06, respectively. The Strehl ratios for the sample with defects and the defect-free sample can be calculated as 0.86 and 0.94 by comparing them with the standard sample at that diameter and focal length. This indicates that defects in the sample affect the optical performance of the sample.

The wave aberration of the sample consists of two components: the wave aberration caused by defects and the wave aberration from the inherent design structure of the sample. By comparing Figs. 6a, and f, we can identify the influence of different defects on the phase modulation of the metalens. The three kinds of defects on the sample surface shown in Fig. 6a correspond to the penetration of the surface silicon layer, scratches, and stains on the substrate. Both can be identified in the wave aberration shown in Fig. 6f. Penetration and scratches often being localized in an area, while surface dirt on the sample can have an impact across a wide range. In Fig. 6i, the wave aberration of the defect-free sample displays a ring-shaped structure corresponding to the locations of 0-2π phase jumps in the metalens design.

Figures 6g and j show the Zernike expansion of the aberrations. The terms 9th, 16th, and 25th correspond to the spherical aberration of the metalens. Comparing the coefficients of the 9th term in the Zernike expansion in Figs. 6g, and j, defects on the sample surface introduce a larger spherical aberration to metalens. This is primarily due to surface dirt covering a larger area of the sample, while more localized processing defects and scratches do not contribute significantly to the lower-order Zernike modes. Figure 6j shows that the ring-shaped wave phase differences induced by the inherent structure of the sample predominantly contribute to the 5th, 7th, 16th, and 17th terms of the Zernike polynomial, with the 16th term corresponding to high-order spherical aberration.

**Fig. 7:**
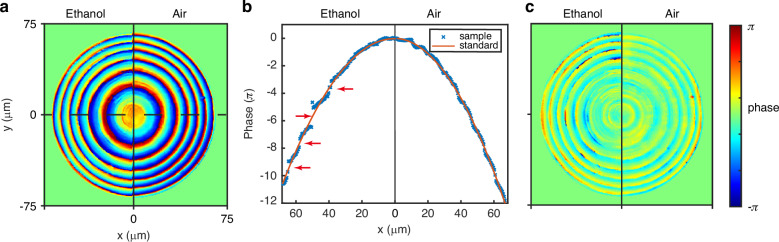
Phase retrieval results of the metalens at a wavelength of 405 nm. **a** Phase distribution, **b** unwrapped phase distribution at the center of the sample, and **c** wave aberration of metalens working in ethanol (left) and air (right)

Lately, metalens operating in different working mediums have garnered widespread attention^35,36^. Here, we measure the phase of a sample that works both in air and ethanol. The measurement system for the metalens in ethanol is illustrated in Fig. S16. Figure 7a shows the phase distribution of the sample in ethanol (left) and air (right). The phase distribution of the sample seems to exhibit noticeable differences in different mediums. To further observe the differences in phase distribution in different mediums, the unwrapped phase distribution along the horizontal centerline of the sample in Fig. 7a is illustrated in Fig. 7b. In comparison to the air environment, immersion in ethanol results in regions of discontinuity in the unwrapped phase of the sample. This indicates that some of the meta-atoms’ phase modulation has changed due to the difference in the working medium. Changes in the working medium affect the resonance of metaatoms with large phase modulation, resulting in a reduction in phase modulation. Therefore, the phase distribution of the sample in ethanol changes compared to that in air. Figure 7c illustrates the aberration distribution. Due to the altered phase modulation of metaatoms, the aberration of the sample increases in ethanol. Note that over time, the changing of metalens’ performance working in different mediums has been widely studied, but the underlying reasons were previously unknown. For the first time, we have measured the changes in the phase modulation of the metalens due to variations in the working environment.

## Discussion

In this work, we have established a multi-distance phase retrieval system for measuring the metalens phase. Our system, which extracts phase from intensity maps, has minimal requirements for laser coherence, enabling its application across various mediums and wavelengths. With this method, we measured the phase of two metalens with the sample parameters working at NIR. After retrieving the phase, all other optical properties can be determined, such as the focal length and wave aberration. Furthermore, we measured the variation in phase modulation of the metalens when immersed in ethanol compared to air. For this system, we introduce a simulation-based convergence and robustness analysis paradigm, which can address different requirements for measurement system stability and data in the case of different samples.

## Materials and methods

### Design and fabrication of 405 nm metalens

The commercial simulation software COMSOL Multiphysics® is used to design and simulate the meta-atoms of the metalens. Gallium nitride (GaN) is chosen as the base material of meta-atoms with the shape of the cylindrical nanopillar on a sapphire substrate. The diameter of the nanopillars varies across the metalens. Fig. S17 shows the transmission spectra and phase shift of different nanopillar sizes. The refractive index of the sapphire substrate is set to 1.77, and the refractive index of GaN at the working wavelength is 2.42. The work wavelength of this metalens is 405 nm. This metalens is a square metalens with a side length of 150 μm. The designed focal length is 900 μm.

The fabrication process of the 405 nm metalens is introduced as follows. A double-polished sapphire substrate is prepared to grow an 800-nm-thick GaN layer by metalorganic chemical vapor deposition (MOCVD). For the high aspect ratio etching, a 400-nm-thick SiO_2_ layer is deposited as the hard mask layer by plasma-enhanced chemical vapor deposition. A positive tone electron-beam resist diluted ZEP520A (ZEP-520A:ZEPA = 1:3) is spin-coated onto the GaN substrate, and the layout is defined by an electron-beam lithography process. A 40-nm-thick Cr layer is deposited by the electron-gun evaporator and acts as a metal etching mask for the SiO_2_ layer. N,N-Dimethylacetamide (ZDMAC) solution is used for the lift-off process. The layout is transferred to the SiO_2_ layer by reactive ion etching (RIE). The high aspect ratio GaN nanopillars are produced by the inductively coupled plasma reactive ion etching (ICP-RIE) with the SiO_2_ hard mask layer. The remaining SiO_2_ layer is removed by the buffered oxide etch (BOE) solution, and the final sample is obtained.

### Design and fabrication of NIR metalens

Amorphous silicon (α-Si) is chosen as the base material of meta-atoms with the shape of the crescent nanopillar on a silica substrate. The rotation angle of the nanopillars varies across the metalens to acquire the geometric phase. The refractive index of the silica substrate is set to 1.45, and the refractive index of amorphous silicon at the working wavelength is 3.43. The work wavelength of this metalens is 1560 nm. This metalens is a circular metalens with a diameter of 90 μm. The designed focal length is 220 μm. For fabricating the NIR metalens, a 327 nm-thick α-Si film is grown on the glass substrate. A 22 nm-thick Cr layer is deposited as a metal mask by an electron beam evaporator. A PMMA layer is spin-coated on the prepared substrate for patterning. The layout is transferred to the PMMA resist by the electron beam lithography process and developed in MIBK/IPA solution. After the resist development, the Cr and Si are etched by inductively coupled plasma. Later, the remaining Cr layer is removed by the chromium etchant for 10 min. Finally, the α-Si-based NIR metalens is obtained.

### Supplementary information（please use the attached PDF file）

Supplementary Information
