# Supplementary material for: Metalenses phase characterization by multi-distance phase retrieval
Source: Light Sci Appl. 2024 Aug 6;13:182. doi: 10.1038/s41377-024-01530-1 (PMC11303724; doi:10.1038/s41377-024-01530-1)
Supplement: Supplementary file 1 — Supplementary Information [file 41377_2024_1530_MOESM1_ESM.pdf]

Supplementary Information for

Metalenses phase characterization by multi-distance phase retrieval

Bowen Liu[1,#], Jialuo Cheng[2,#], Maoxiong Zhao[1,*], Jin Yao[2], Xiaoyuan Liu[3], Shaohu Chen[1], , Lei Shi[1,4,5,6], Din Ping Tsai[2,3,7], Zihan Geng[8,*], and Mu Ku Chen[2,3,7,*]

[1]State Key Laboratory of Surface Physics, Key Laboratory of Micro- and Nano-Photonic Structures (Ministry of Education) and Department of Physics, Fudan University, 200433 Shanghai, China

[2]Department of Electrical Engineering, City University of Hong Kong, Kowloon, Hong Kong SAR, China

[3]State Key Laboratory of Terahertz and Millimeter Waves, City University of Hong Kong, Kowloon, Hong Kong SAR, China

[4]Institute for Nanoelectronic Devices and Quantum Computing, Fudan University, 200438 Shanghai, China

[5]Collaborative Innovation Center of Advanced Microstructures, Nanjing University, Nanjing, 210093 Jiangsu.China

[6]Shanghai Research Center for Quantum Sciences, 201315 Shanghai, China

[7]Centre for Biosystems, Neuroscience, and Nanotechnology, City University of Hong Kong, Kowloon, Hong Kong SAR, China

[8]Institute of Data and Information, Tsinghua Shenzhen International Graduate School, Tsinghua University, Shenzhen, Guangdong 518071, China.

[#]These authors contributed equally to this work

*Correspondence: maoxzhao@fudan.edu.cn; geng.zihan@sz.tsinghua.edu.cn; mkchen@cityu.edu.hk

1. Phase retrieval algorithm for MDPR system

Initially, generate a random sample-plane field distribution $E^{s,k}$, where s is the sample plane, k is the number of iterations. Use the angular spectrum propagation method to calculate the field distribution corresponding to the given sample-plane field distribution at that position d_i.

$$E_i^f = F^{-1}e^{ik_z d_i}F(E^{s,k}) \tag{1}$$

where f represents the far-field, F is the Fourier transform, k_z is the propagation direction wavevector.

Extract the phase of the calculated field distribution E_i^f at position d_i, combine it with the measured intensity distribution I_i, and obtain the updated far-field field distribution.

$$\varphi_i = arg\left(E_i^f\right) \tag{2}$$

$$E'^f_i = \sqrt{I_i}e^{i\varphi_i} \tag{3}$$

Subsequently, the updated N far-field light field distributions are backward propagated to the sample plane position.

$$E_i^s = F^{-1}e^{-ik_z d_i}F\left(E'^f_i\right) \tag{4}$$

To balance the information across the various intensity distributions, the obtained N sample-plane field distributions are averaged.

$$E''^{s,k} = \frac{1}{N}\sum_{i=1}^{N} E_i^s \tag{5}$$

To enhance the iterative convergence of the algorithm, double-feedback introduces data from the k-1, k-2 iterations when $k > 2$

$$E^{s,k+1} = (1 + a + b)E'^{s,k} - aE^{\,s,k-1} - bE^{\,s,k-2} \tag{6}$$

Through experimentation with actual data, it was found that parameters a and b exhibit the best convergence when their values are between 0.7 and 0.75.

The 'Double-feedback' step (6) in the algorithm is primarily aimed at enhancing the algorithm's noise tolerance and convergence speed. The selection of parameters a and b is influenced by the measurement conditions, especially influenced by the noise level and translation stage accuracy during measurements. We simulated the distribution of MSE between the phase retrieval results and the true values under different values of a and b for the same measurement conditions, as shown in **Fig.S1**. It can be observed that within a certain range (depicted by the white region in **Fig.S1**), the values corresponding to a and b can lead the algorithm to converge near the true values.

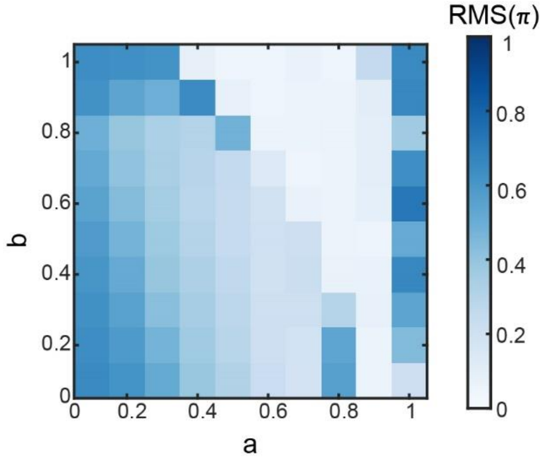

Fig. S1: The sampled results of the same metalens phase distribution at different sampling intervals. The noise level is 0.1, and the uncertainty of the displacement stage is 1 μm after 80 iterations.

Fig.S2 illustrates the relationship between the MSE and the total scanning field length and iteration times. It can be observed that when the scanning field length is insufficient, the MSE remains unchanged with iteration number increases, indicating the algorithm is trapped in a local minimum. However, when the scanning field length is sufficiently long, the algorithm converges to the true value within ten iterations, avoiding the local minimum.

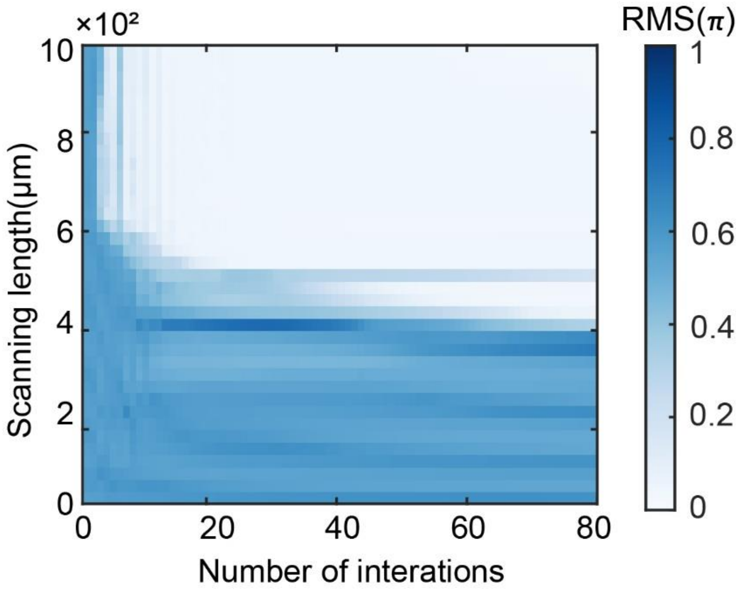

Fig. S2: Relationship between RMS error, iteration number, and scanning field length with 30 pictures used for phase retrieval.

In practical industrial applications, the convergence speed of the iterative algorithm is crucial. Therefore, we need to introduce more information to assist the algorithm in converging faster. Compared to traditional lensless imaging systems, our scanning system, based on a microscopic imaging system, allows for the capture of intensity distribution at the sample plane. Inspired by Kalman filtering, we employed a method of controlling weights ω to achieve balancing the intensity distribution obtained from measurements with the intensity distribution obtained through iteration in **Eq.S5**

$$\Phi^s = arg(E''^{sk})) \tag{7}$$

$$E'^{sk} = \left(\omega\sqrt{I_0} + (1-\omega)|E''^{s,k}|\right)e^{i\phi^s} \tag{8}$$

By adjusting weights ω, we balance the direct measurement results (intensity distribution at the sample plane) with the indirect measurement results (intensity distribution obtained during the algorithm iteration process). This introduces measurement information at the sample plane, making it closer to the target value and allowing the algorithm to converge faster. Qualitatively, as the iteration progresses, we know that the iterative result will continuously approach the true value. Therefore, we choose to balance the iterative field intensity at the sample plane with the measured intensity using linearly changing weights ω from 1 to 0.

Fig.S3 shows the variation of MSE to the number of iterations across varying scan lengths, depicting both the algorithm's performance in its raw form and its enhancement

with Kalman filtering, as depicted in **Fig.S3(a)** and **Fig.S3(b),** respectively. After introducing the Kalman filtering step, the iteration was accelerated by 1/3, reducing from 15 to 10 iterations. Additionally, with the introduction of sample intensity, the MSE decreases monotonically during the iteration process, indicating that the iterative algorithm is more stable.

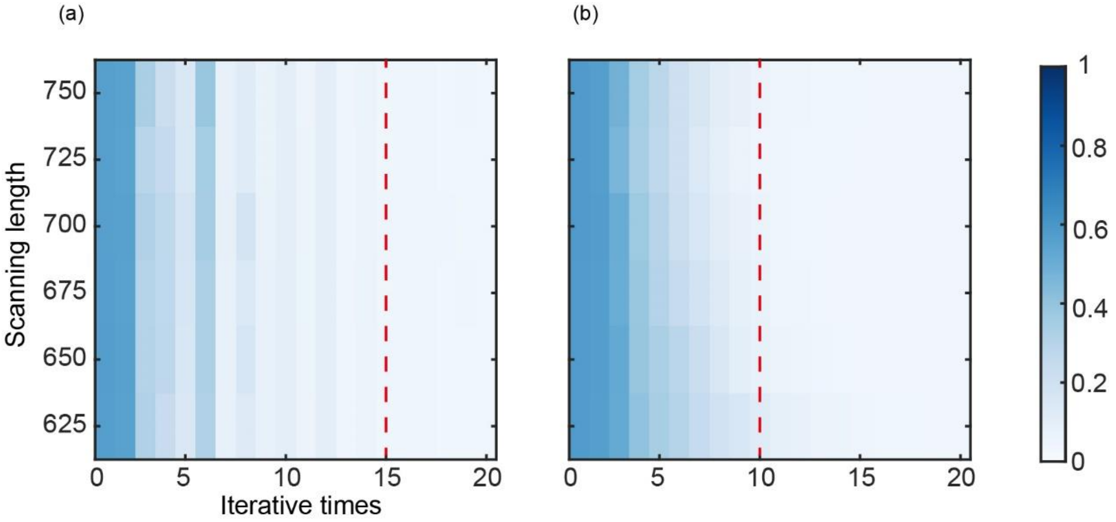

Fig. S3: The variation of MSE with the number of iterations and the total length of the scan. (a) Without Kalman filtering (b) With Kalman filtering.

Due to the focusing effect of the metalens on the light beam, the intensity distribution behind the sample undergoes drastic changes. One case is when the scanning length is compatible with the focal length. When we require the optical path to capture clear images near the sample surface, images closer to the focus may be overexposed. Another case is that under immersion conditions, multiple interfaces of liquid, container walls, and air reflections introduce coherent noise into the optical path, typically appearing as bright speckles. The intensity measurement here is erroneous, so such erroneous intensity information should not be introduced into the iteration process. For these reasons, we introduce a mask function M in updating the far-field intensity in **Eq.S3** and update it as

$$E_i'^{f} = \left(M\sqrt{I_i} + (1-M)\left|E_i^f\right|\right)e^{i\phi_i} \tag{9}$$

Where

$$M = \begin{cases} 0, & I_i = I_{max} \\ 1, & else \end{cases} \tag{10}$$

M is the Mask function for the selected region. By introducing M, we address the

impact of coherent noise in the optical path under immersion conditions, as shown in **Fig.S4**.

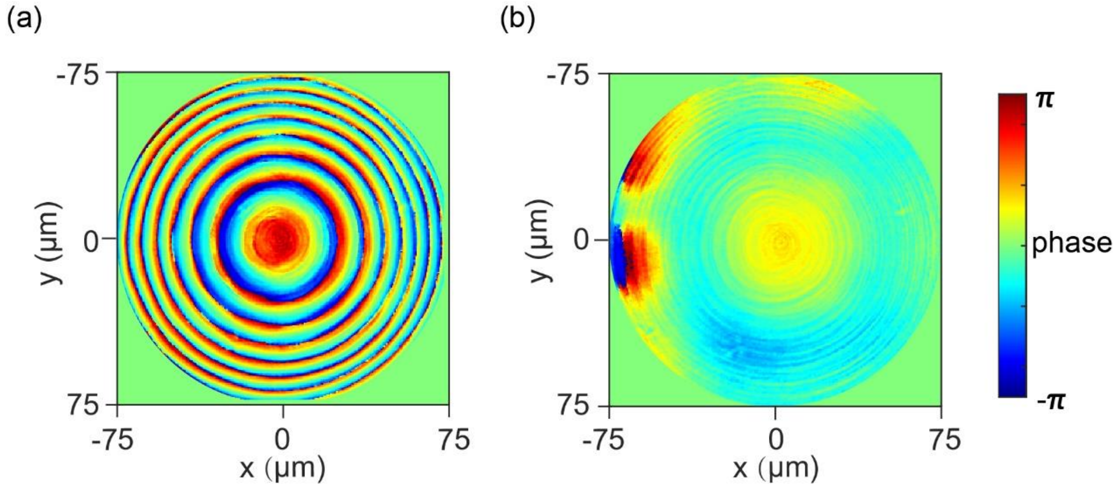

Fig. S4: Phase retrieval results of the immersion system. (a) With Mask function, (b) Without Mask function.

Another region where intensity updating is unnecessary is when the intensity values obtained through iteration are very close to the measured intensity values themselves. This is because, due to the presence of CCD noise, The true intensity falls within a range around the measured value $[I_i - \Delta I, I_i + \Delta I]$ where ΔI is associated with the inherent properties of the CCD and its integration time. So M is determined as

$$M = \begin{cases} 0, & I_i = I_{max} \; or \; \left|E_i^f\right|^2 - I_i \in [-\Delta I, \Delta I] \\ 1, & else \end{cases} \tag{11}$$

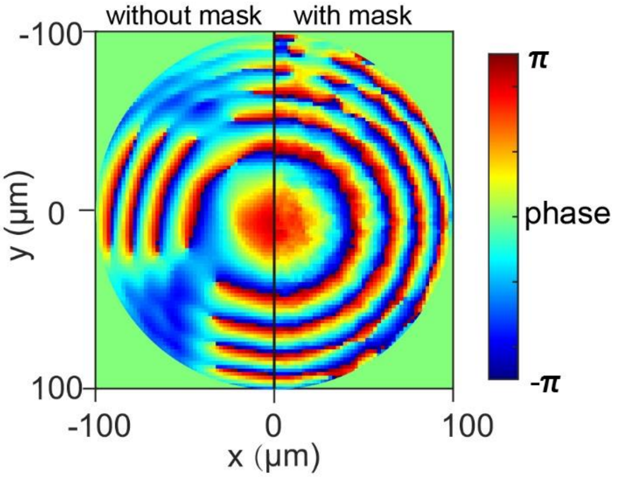

Fig. S5: Phase retrieval results of elliptical metalens NIR metalens with

high noise condition measurement.

The noise range ΔI can be obtained by continuously taking photos without incident light and calculating the fluctuation of the background noise distribution. With the use of such a region selection mask function M, we achieved phase retrieval of elliptical metalens under high-noise conditions, as shown in **Fig.S5**. After using the mask function in (11), we can observe the elliptical phase fringes of the metalens. Hence, it's imperative to identify images affected by these incidental disturbances and exclude them from the phase retrieval process.

All the optimizations we discussed so far are based on optimizing the noise of the measurement system itself, which can accelerate the convergence speed of the algorithm and enhance its robustness. However, in actual experimental processes, we found that occasional errors during measurements can greatly affect the convergence of the algorithm. These occasional errors may arise from the optical platform's vibration or the introduction of ambient light. Especially in immersion conditions, vibrations from other instruments on the same optical platform can affect the smoothness of the liquid surface. External occasional disturbances can lead to significant errors in the intensity or position of an image during the scanning process, affecting the convergence and accuracy of the algorithm. Hence, it's imperative to identify images affected by these incidental disturbances and exclude them from the phase retrieval process.

We realized that during the iterations, we achieved a balance across intensity measurement in all positions at the sample plane. Since incidental disturbances occur only in a few images, the corresponding sample surface field distributions for these images should exhibit significant differences compared to those for images unaffected by the disturbances. By calculating the field distributions between the sample surfaces corresponding to different images, we can eliminate the images affected by disturbances. After the iterations have stabilized, compute the RM deviation between E_i^s .

$$RMS(i,j) = \left|E_i^s - E_j^s\right|^2 \tag{12}$$

A simulated result of the RMS deviation distribution is shown in **Fig.S6**. We introduced arbitrary disturbances to the measurements of position and intensity at five locations in the simulation, causing them to deviate from the overall measurement results. Our method can filter out images corresponding to larger RMS values numbered 2, 9, 22, 23, and 27, which also means that after removing these images, the system can retrieve

the phase precisely.

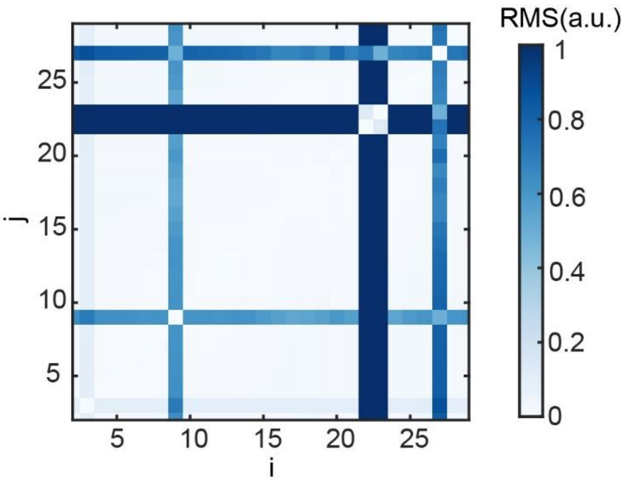

Fig.S6: The near-field field distributions corresponding to different far-field positions. i, j are the image numbers.

The time complexity of the phase retrieval algorithm needs to be considered in two parts: the time complexity of the iteration process and the time complexity of angular spectrum propagation. For iterating T times using M images, the time complexity is $M \times T$. The time complexity of the angular spectrum propagation process mainly comes from the calculation of the k-space distribution of the optical field sized N using 2-D FFT, which has a time complexity of $N^2 \log N$. Therefore, the total time complexity of the algorithm is $M \times T \times N^2 \log N$. On a PC configured with AMD Ryzen 7 5800H with Radeon Graphics and 16GB RAM, the time for phase retrieval of the NIR sample using 30 images sized 256×256 for 100 iterations in non-parallel conditions is 13 seconds.

2. The accuracy of the MDPR system and its consistency with off-axis interferometry.

2.1 consistency with off-axis interferometry.

The consistency of measurement results across different methods is a key issue in metrology. We used off-axis interferometry and the MDPR system to measure the phase of the same metalens. The difference in aberration between the two methods is shown in **Fig.S7(a)**, with a RMS deviation of 0.16.

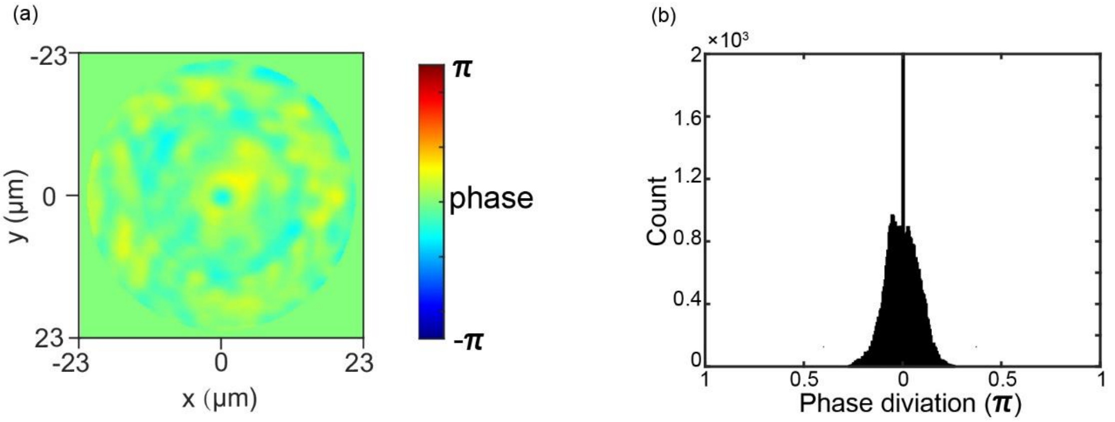

Fig. S7: Difference between off-axis interference and MDPR wave aberrations. (a) Difference of aberration between two methods, (b)statistics of (a).

2.2 The accuracy of the MDPR system

We calibrated the phase accuracy of our method using standard samples, and the result is shown in **Fig.S8**. We scanned a pinhole in 532 nm without internal phase modulation inside, so all phase fluctuations in the measurement result are due to measurement errors. Using a 3 mm scan field data, the standard deviation of the phase retrieval obtained is 0.02 rad, which corresponds to a path length error of 1.6 nm (0.3% λ).

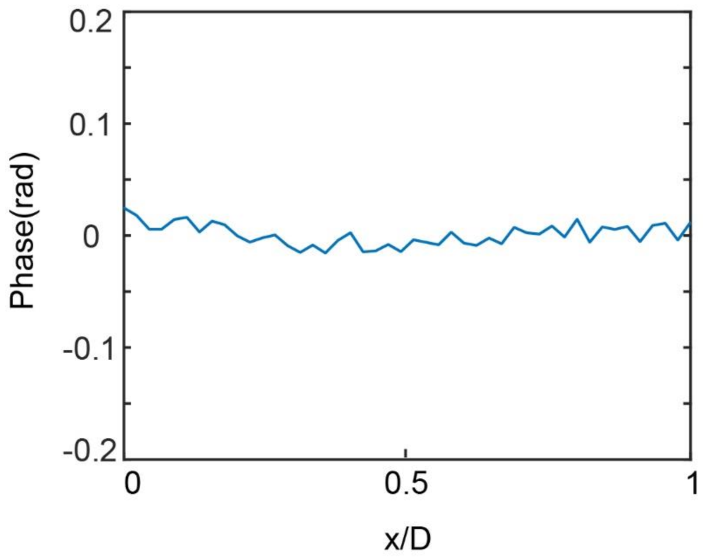

Fig. S8: Phase retrieval results for the standard sample. *D* is the normalization coefficient for length.

2.3 The impact of the broadening of the incident light spectrum

Fig.S9 shows the spectrum of the illumination laser, with the bandwidth $\Delta\lambda$ of 10 nm.

Its coherence length is $\frac{\lambda^2}{\Delta\lambda} = 243\ \mu m$. Under this condition, it is difficult to use interferometric methods to extract the phase distribution. We further simulate the effect of light source coherence on phase retrieval. We calculated the optical field distribution of the sample with the same phase response at different wavelengths, weighted by the measured spectral intensity distribution, and obtained the three-dimensional optical field distribution under partial coherence. Fig.S8 shows the phase retrieval results of the partly coherent optical field, and its standard deviation from the reference value **Fig.S10** within the sample region is 0.06 rad, which indicates that the coherence of our light source is sufficient to meet the requirements for phase retrieval.

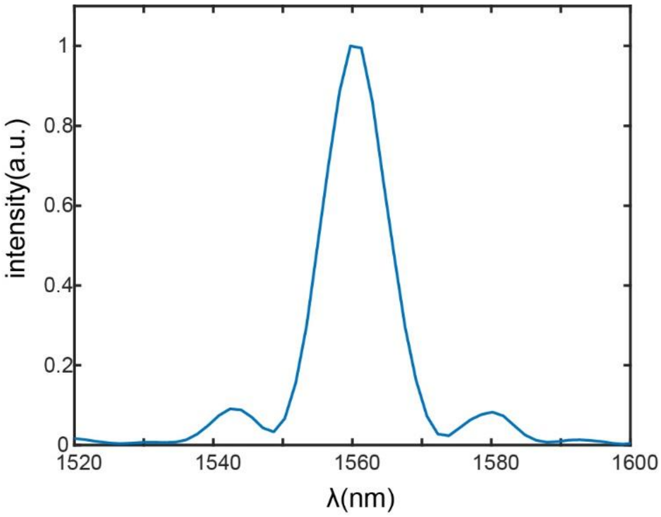

Fig. S9: The measured spectrum at a laser wavelength of 1560 nm.

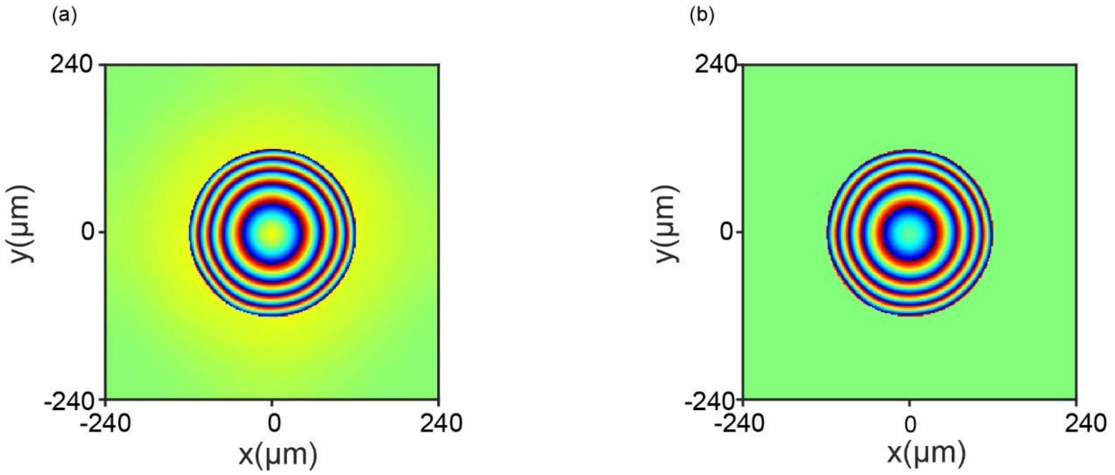

Fig. S10: Phase retrieval results for partially coherent light. (a) Retrieval results. (b) Standard value.

3. Measurement system.

The performance of the fabricated NIR metalens is verified by an optical microscope, scanning electron microscope (SEM), and a homemade light field scanning measurement system. The optical image and the SEM image are shown in **Fig.S11**. The incident laser with a wavelength of 1560 nm is generated by a supercontinuum white light laser and an acousto-optic tunable filter (AOTF). A beam expander is used to make the spot size of the incident light much larger than the size of the NIR metalens. The light field scanning measurement system is shown in **Fig.S13**. The polarization state of the incident laser beam is converted to a circular polarization state by using a linear polarizer and a quarter-wave plate. The objective (Mitutoyo 50×, NA = 0.55) is used to collect the focused spot from the metalens in transmission. Another set of a linear polarizer and a quarter-wave plate is used to select the modulated light of the NIR metalens. The green dashed box includes the objective, the linear polarizer, the quarter-wave plate, and an NIR camera (Hamamatsu, C10633-23) are mounted on a motorized stage (Newport, GTS70) to scan and capture the light intensity profile on the z-axis.

Fig.S12 shows the sample image during the scanning. Due to the sample relying on cross-polarization for field focusing, non-sample regions exhibit dark noise after polarization separation, which is zero after subtracting the background.

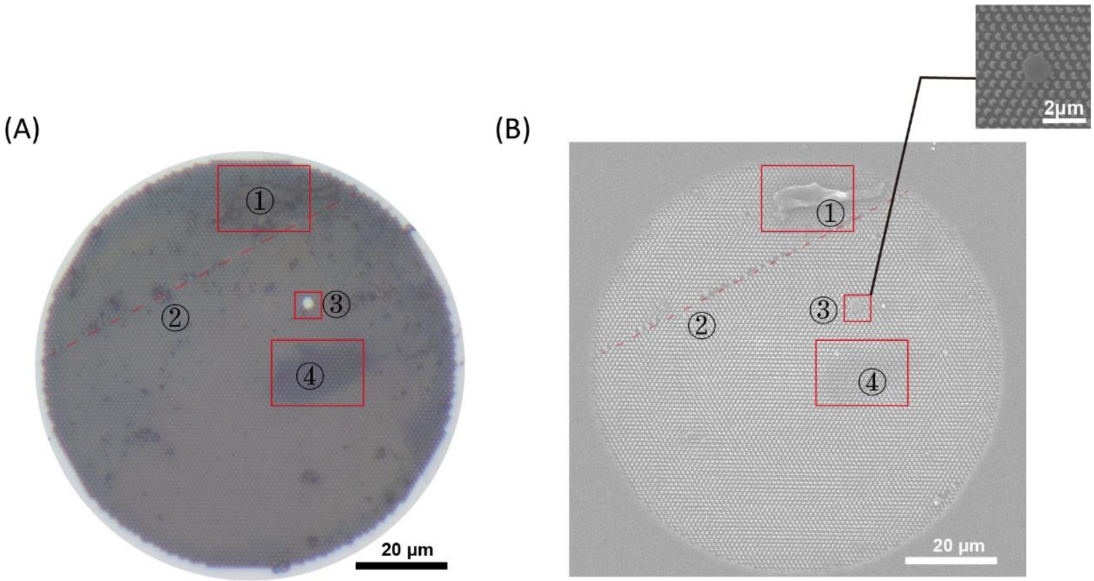

Fig. S11. The optical image (A) and the SEM image (B) of the fabricated NIR metalens.

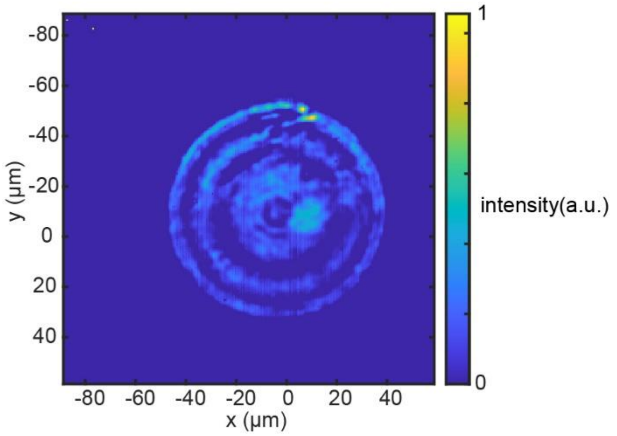

Fig. S12. Sample surface image during the scanning of the infrared sample.

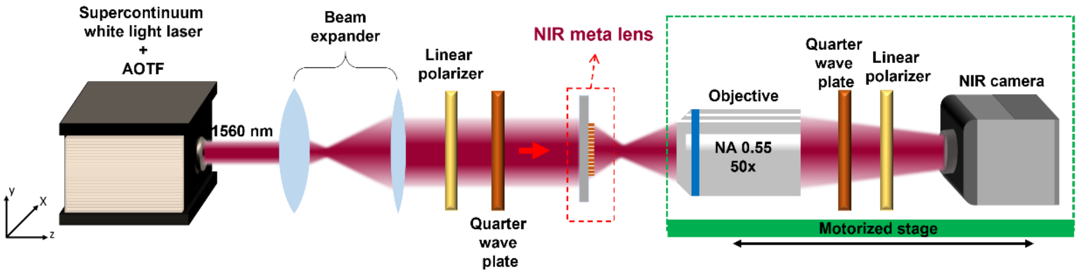

Fig. S13. The light field scanning measurement system for NIR metalens.

Due to factors such as CCD dark noise and readout noise, there are discrepancies between the recorded light intensity data by the CCD and the actual received light intensity. This discrepancy is propagated through the iterative algorithm to the obtained phase. In the experiment, we measured the distribution of CCD noise, as shown in **Fig.S14**, which can be considered to follow a Gaussian distribution. The intensity values from the camera range from 0 to 255.

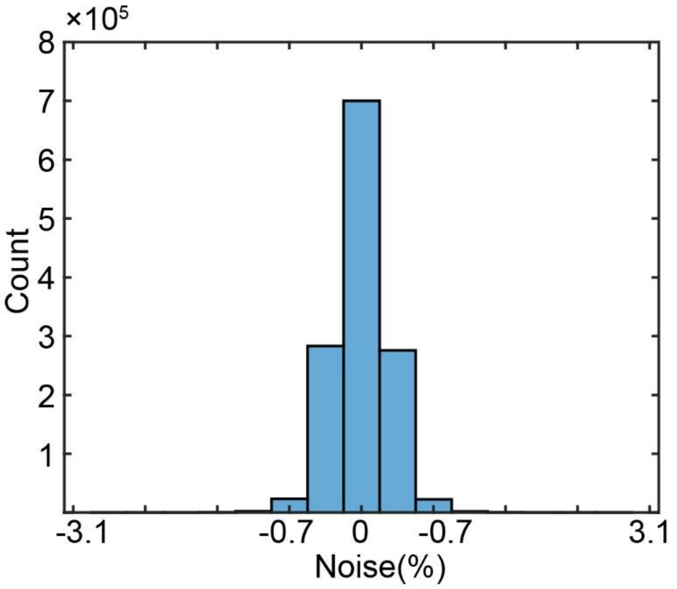

Fig. S14: Statistical data of the noise

4. Zernike analysis of defects in the NIR sample

In general, we are mainly concerned with the first 36 terms of the Zernike expansion corresponding to the sample wavefront aberration. These terms represent low-frequency information and characterize the global properties of the sample. However, defects on the sample are often localized, a single defect on the sample surface may correspond to many terms in the Zernike expansion. Depending on the origin of defects, defects on the sample can be categorized into dust, stain, scratch, and manufacturing defects (hole). For a given sample, further analysis of the impact of defects on the Zernike expansion coefficients of the sample can be conducted by setting the wavefront aberrations to zero in other regions and retaining only the wavefront aberrations in the specified region, then analyzing the influence of the wavefront aberrations from the specified origin. Note that setting the wavefront aberrations to zero means setting the phase difference to zero, not setting the phase to zero. A zero value for the wavefront aberrations indicates that there is no deviation from the standard phase at that position, meaning the phase at the zeroed position is equal to the standard phase. Dust and stain often cover larger areas and contribute to the first 36 Zernike coefficients. Scratches and manufacturing defects cover smaller areas, and their corresponding phase differences contribute to Zernike coefficients beyond the first 36, often not considered, as shown in **Fig.S15**. In addition to defects in the sample, errors in the processing and design of the sample itself can also introduce phase differences. This part contributes to the majority of the phase differences in most of the areas. The contributions of dust and stain areas to the Zernike expansion are shown in **Fig.S15**(e) and **Fig.S15**(f). By comparing the Zernike expansion coefficients of the phase aberrations from different sources with the total Zernike expansion coefficients, the 29th Zernike expansion coefficient of the sample is contributed by the stained part, while the 11th, 14th, 21st, 27th, 30th, and 32nd coefficients are contributed by the dust part. After removing the four local wavefront aberrations mentioned above, the rest of the coefficients come from processing and design errors, as shown in **Fig.S15**(a) and **Fig.S15**(b). It is worth noting that even for identical defects, their contribution to the Zernike expansion coefficients can vary depending on their location on the sample.

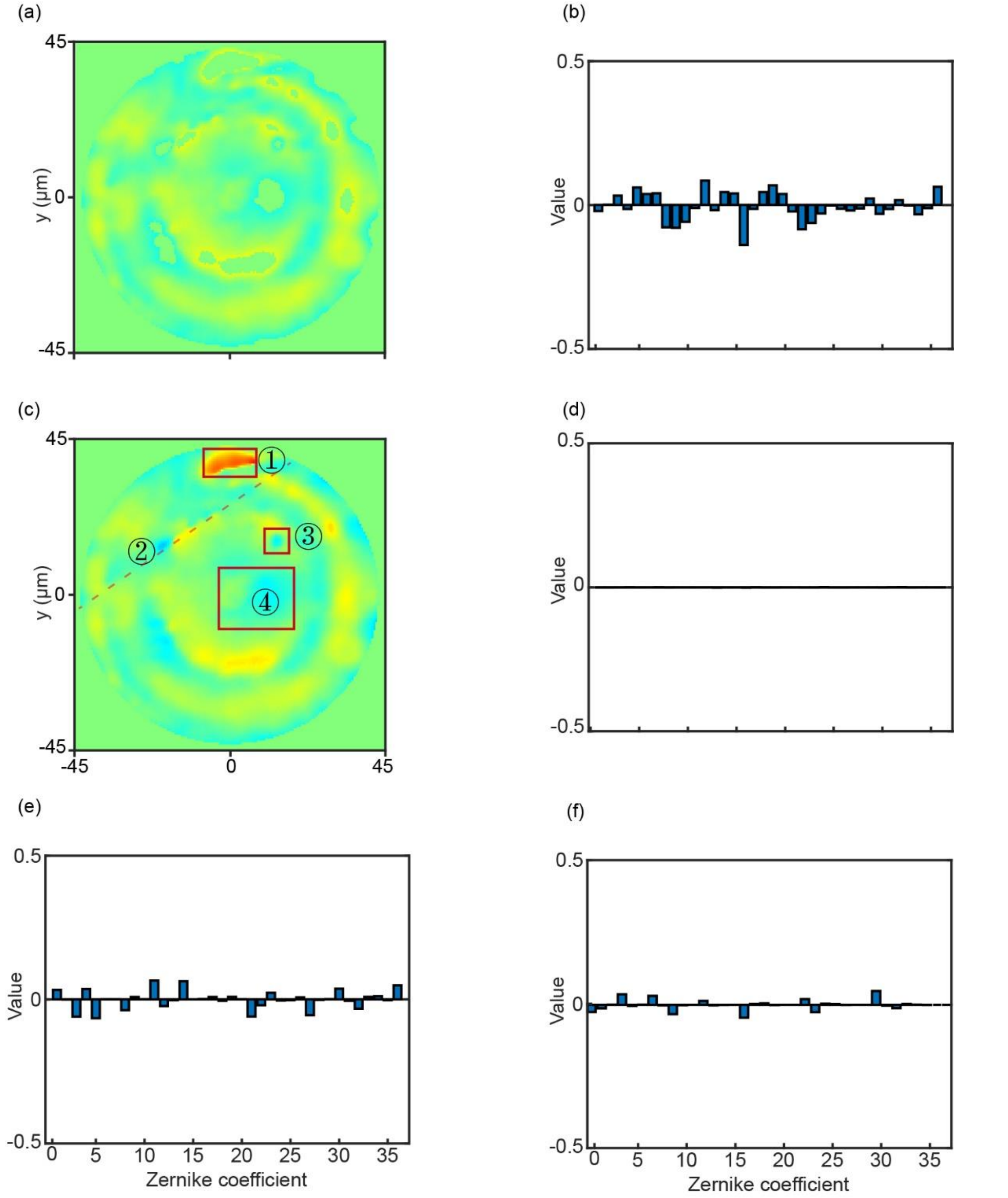

Fig. S15: Zernike expansion coefficients for wave aberrations from different sources. (a) Distribution of wave aberrations due to processing and design errors. (b) Zernike expansion of aberration in (a). (c) Total aberration of the sample. (d)-(f) Zernike expansion of aberrations from hole(region ③),dust(region ①),and stain(region ④) respectively.

5. The scanning field measurement optical path for metalens in different mediums

The beam size of the incident light beam is expanded by a beam expander. The beam size is about 3 mm, which is sufficiently large compared to the sample size. Therefore,

it can be assumed that the incident light in the sample region is collimated. Thus, the measured phase distribution is entirely due to the modulation of the sample.

the metalens is placed on the wall of the transparent water tank. The light field scanning measurement system is shown in **Fig.S16**. We placed the metalens in the air (**Fig.S16 (a)**) and immersed it in ethanol absolute (**Fig.S16 (b)**), respectively. The light field intensity distribution of the metalens was collected in these two environments.

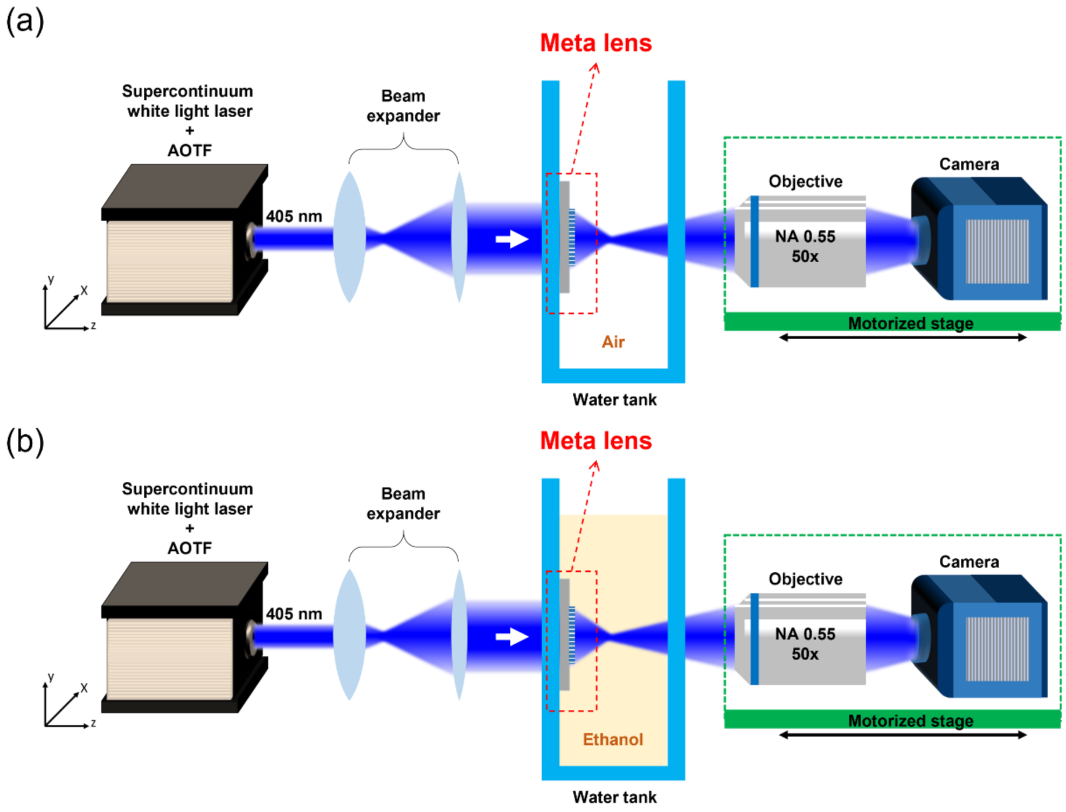

Fig. S16: The light field scanning measurement system. (a) The metalens is in the air. (b) The metalens is immersed in ethanol absolute.

6. The phase distribution of the sample in air and ethanol.

As shown in **Fig.S17(a)**, due to the variation in the scattering cross-section of the metaatoms, the phase modulation of metaatoms with larger radii is more significantly affected by the working medium. This results in the actual phase coverage of the metalens not reaching $0 - 2\pi$, as shown in **Fig.S17.(b)**. Therefore, in the unwrapped phase distribution, at the phase folding positions, the position of the maximum phase in the previous cycle cannot be seamlessly connected to the phase zero point of the next cycle, leading to phase discontinuities.

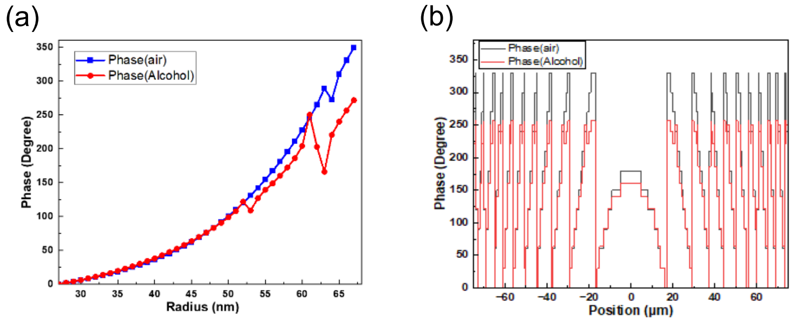

Fig. S17: The effect of changing the working medium on the sample. (a) The modulation of the meta-atom phase in different media as a function of radius (b) The discretized phase distribution profile of metalens. The black line is the phase distribution in air. The red line is the phase distribution in alcohol.

7. The effect of sample tilt on the phase.

In the case of a tilted sample, we can observe the phase variation caused by the sample tilt. As shown in **Fig.S18**, we can still measure the phase distribution of the sample, and the tilt of the sample will be reflected in the phase. Compared to the measurement results without tilt, the tilt of the sample leads to the superposition of phase fringes in the direction of tilt. After subtracting the first three Zernike terms and the corresponding focal length standard sample phase, we can still obtain the wavefront difference of the sample. However, it is important to note that since the sample is not perpendicular to the incident light, what we measure now is the phase distribution of the sample at this angle, which often differs from the phase distribution under normal incidence due to the sample's angular dispersion.

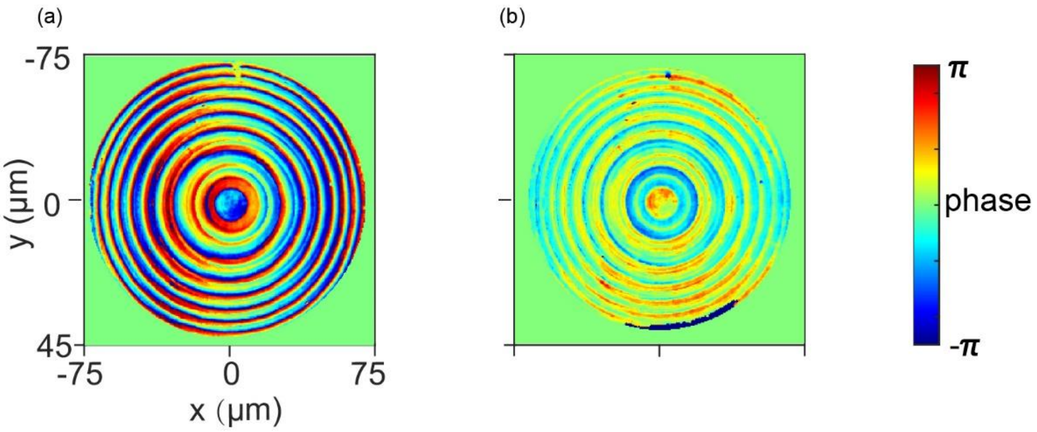

Fig. S18 Phase distribution and wavefront aberration under the tilted sample condition Operating wavelength 405 nm, focal length 875 μm. (a) Phase. (b) Aberration.

8. The influence of scan field length on phase reproduction results

8.1 The influence of scan field length on phase reproduction results

The mean square error does not monotonically decrease with the increase of L, But there is a threshold instead. This is because before reaching the threshold, the phase distribution obtained by the algorithm differs significantly from the standard value, and at this point, the MSE cannot be used to measure the difference between them quantitatively.

The convergence behavior of the algorithm concerning L can be understood as follows: due to the increasing angle of deflection of light at different positions in a metalens with increasing distance from the lens center, the spatial frequency modulation of the light field by the metalens also increases with distance from the lens center. As shown in Fig.S19.(c), for regions with high-frequency optical modulation at the edge of the metalens, the corresponding light field distribution changes dramatically in the z-direction. Rapidly changing intensity in z-direction implies more information, requiring only a short scanning distance to obtain sufficient information. For regions with low-frequency optical modulation at the center of the metalens, the corresponding light field distribution changes smoothly in the z-direction, implying less information and requiring a longer scanning distance to obtain sufficient information. After the scanning total length reaches a threshold, increasing the scanning length only provides redundant information, which helps the robustness of the algorithm but does not affect the convergence. Fig.S19.(d) and (e) show the retrieval results before and after the threshold, indicates before the threshold only regions with high frequency can converge. For the number of images increasing the number of images also introduces more information. Considering both factors, the threshold forms a shape similar to a hyperbola in Fig.S19.(a) and (b).

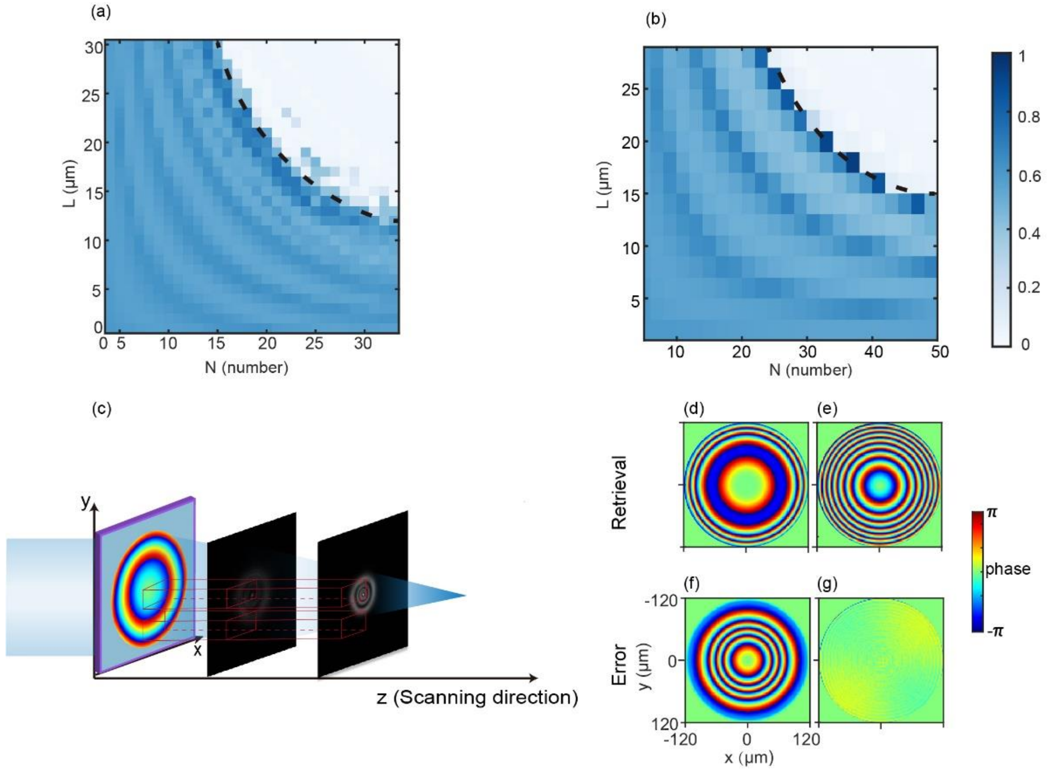

Fig. S19: MSE distribution of MDPR system in metalens measurement with sampling picture number *N* and distance between pictures *L* for sample working at 1550 nm with focal length (a) 0.6 mm and (b) 1 mm. (c) Schematic diagram of light field distribution corresponding to different frequency regions of the sample.

8.2 The influence of the number of pictures on phase reproduction results

Fig.S20 shows the phase retrieval results with a different number of pictures. When the number of images used is less than five, as shown in Fig.S20.(a), an accurate phase distribution cannot be obtained. When the number of images reaches five, the algorithm can converge to the vicinity of the true phase distribution, as shown in Fig.S20.(b), but some noise may still be present. As the number of images increases, the number of noise points will gradually decrease, as shown in Fig.S20.(c).

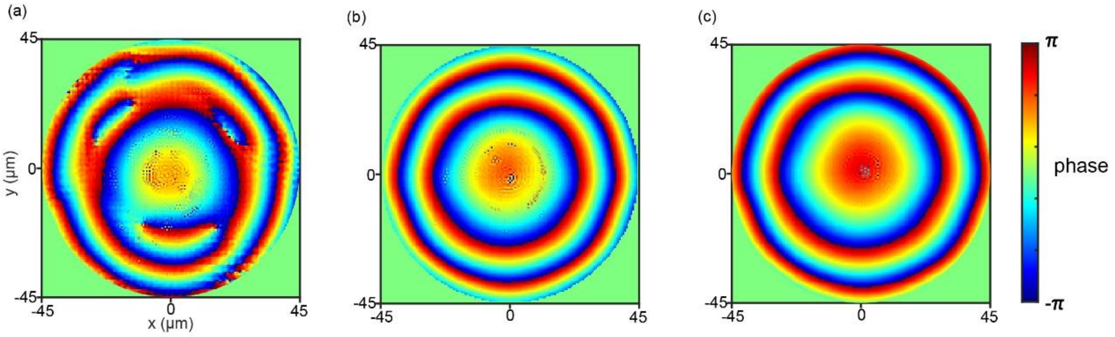

Figure S20: Phase retrieval results of NIR samples with different picture numbers. (a) Results with 4 pictures. (b)Results with 5 pictures. (c) Results with 10 pictures.

9. Phase retrieval for other metasurfaces

Since the implementation of all-optical diffractive neural networks in the THZ regime (*Science 361(6406), 1004-1008, 2018*), optical neural networks have garnered widespread attention due to their high computational speed, low power consumption, and minimal heat generation. To achieve device miniaturization, there is a collective aspiration to imply optical neural network architectures by metasurfaces within the visible light spectrum. As optical neural network chips shrink in size (on the order of hundreds of micrometers), alignment issues for the neural network chip emerge. Deviations in the positions between layers can reduce the accuracy of neural networks and increase system energy consumption, thus limiting the increase in the number of layers in optical neural networks operating in the visible light spectrum, while increasing the number of layers remains advantageous for optical neural networks to enhance accuracy and realize complex functionalities (Light: Science & Applications, 10(1), 196, 2021). At the same time, for each layer of metasurface optical neural network chips, there will inevitably be processing errors. The processing and alignment errors will couple, and it is impossible to directly calibrate the alignment errors of multilayer optical neural networks from a functional perspective. It is necessary to measure the overall optical modulation of the multilayer network and the phase modulation of each layer to decouple the alignment errors of the system from the processing errors.

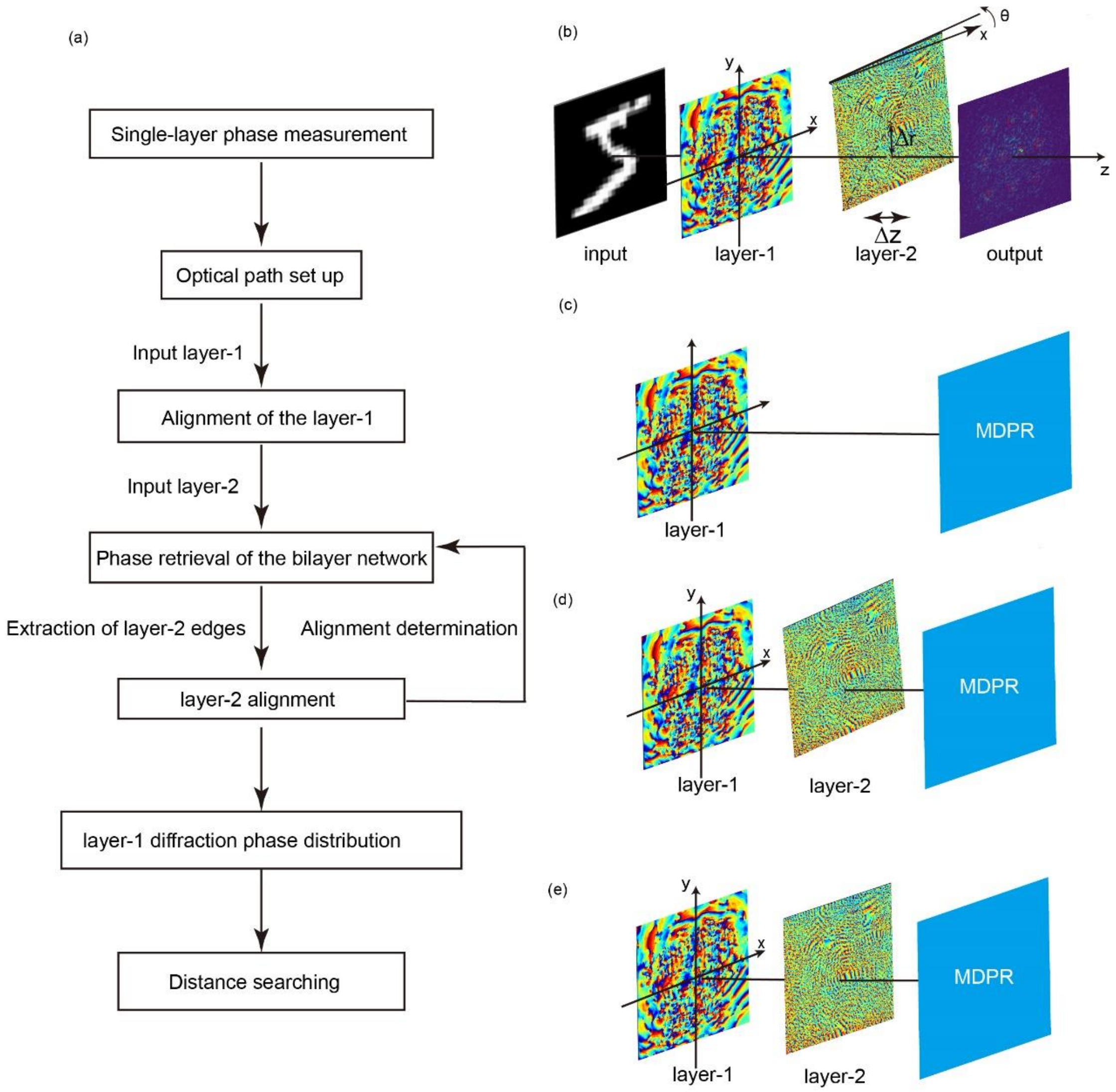

Figure S21: Alignment process diagram for bilayer optical neural network (ONN). (a) Flowchart for alignment. (b) Schematic diagram of ONN working principle and position offset. (c) Phase retrieval for layer-1. (d) Phase retrieval for bilayer ONN. (e) ONN after correcting lateral offset.

For this section, a two-layer metasurface-based optical neural network is used as an example to present the application scenarios of this phase measurement technology. As Fig.S21.(b) shows, there is a certain displacement between two phase-modulating metasurfaces and the input and output layers due to alignment accuracy issues. Taking the layer-2 as an example, it could experience a displacement r relative to the optical axis in the x-y plane, along with a rotation angle θ. Simultaneously, there could be a deviation Δz from the standard value in the z-direction relative to the spacing with the layer-1, while a 2% error in the z-direction position results in a 1% decrease in

classification accuracy.

Our alignment process is illustrated in Fig.S21. (a). Before starting the alignment process, we first measured the phase distributions of the two samples separately as shown in Fig.S22. (a) and (b). While measuring the phase, we can determine the sample's position and its uncertainty. Due to the fluctuations in phase background and resolution of the optical path, the boundaries of the sample fall within the two dashed lines in Fig.S22. (c). Taking half of the width $\Delta x/2$ as the measurement uncertainty of the position, which is 3 μm.

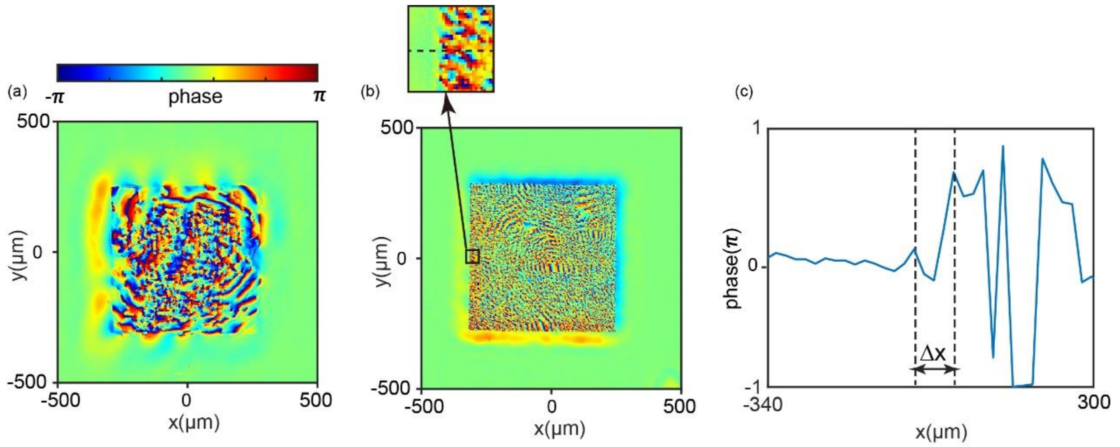

Figure S22: Phase retrieval results for metasurfaces ONN chips. (a) The layer-1, (b) The layer-2 and its detail enlargement. (c) Phase distribution at the dashed line in (b).

Here, we demonstrate a simulation-based alignment process of a double-layer metasurfaces system. During the alignment process, we first align the layer-1 with the optical path. By scanning the intensity distribution, the position of the layer-1 can be obtained, and the layer-1 can be adjusted to the center of the optical path, as shown in Fig.S23. (a). Afterward, we added the layer-2. At this point, the phase distribution is contributed by two parts: the diffraction from the layer-1 and the modulation from the layer-2. Fig.S23. (b) shows the phase modulation of the bilayer optical neural network system measured by MDPR. Therefore, it is difficult to directly determine the precise boundary position of the layer-2. Since the distance between layer-1 and layer-2 is much larger than the size of the metasurfaces (about 100 times), the contribution of diffraction from layer-1 at the position of layer-2 is low-frequency. By removing the contribution

of the first-layer diffraction through high-pass filtering, the boundary of the second-layer metasurface can be obtained within a single pixel, which indicates that high-pass filtering will not cause smoothness in the edge. As Fig.S23. (c) shows offset in xy-plane, $r = (-103{,}91)$ μm and a clockwise rotation $\theta = 2.18°$.

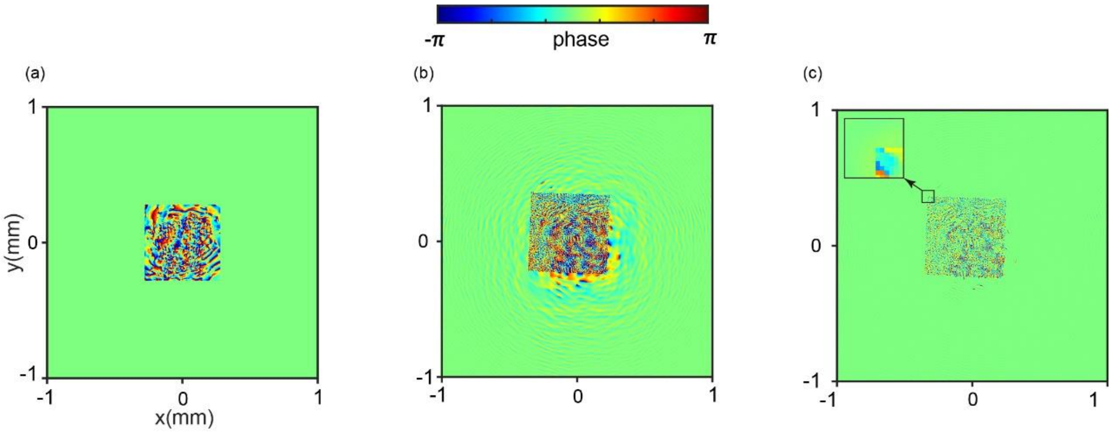

Figure S23: Simulation results of the 2 layers optical neural network. (a) Place the layer-1 in the center of the optical path. (b)Phase retrieval results for the bilayer sample. (c)High-pass filtering results of (b).

After obtaining the offset of layer-2 in the xy-plane, its center can be shifted to the center of the optical path and rotated to align with the optical path. The phase of the two plates can then be measured again using the MDPR method to confirm that the two plates are aligned with the optical path in the xy-plane, as shown in Fig.S24. (a). Otherwise, the alignment process described above will be repeated. Then, by removing the phase modulation of layer-2 from the overall phase distribution, the phase distribution of the diffraction field of layer-1 at this position can be obtained as shown in Fig.S24.(b). Due to alignment accuracy issues, some high-frequency information may remain after removing the phase distribution of the layer-2 as shown in Fig.S24.(b). This high-frequency information can be removed by using a low-pass filter, as shown in Fig.S24. (c). Through the forward propagation of the phase distribution of layer-1 in Fig.S23. (a), the phase distribution at different positions can be computed. Then, calculate the standard deviation between these phase distributions and Fig.S24. (c), the position with the lowest STD corresponds to the separation between the two layers of samples returned by the algorithm. The deviation of the distance between layer-1 and layer-2 from the standard value (preset deviation) is 10 μm. It is worth noting that, relative to layer 1, the position of the layer-2 has already entered the far-field diffraction

region (40 mm), where the distribution of the phase is not sensitive to small perturbations in the z-direction, a 0.02% offset in the z-direction corresponds to a negligible deviation that does not impact functionality.

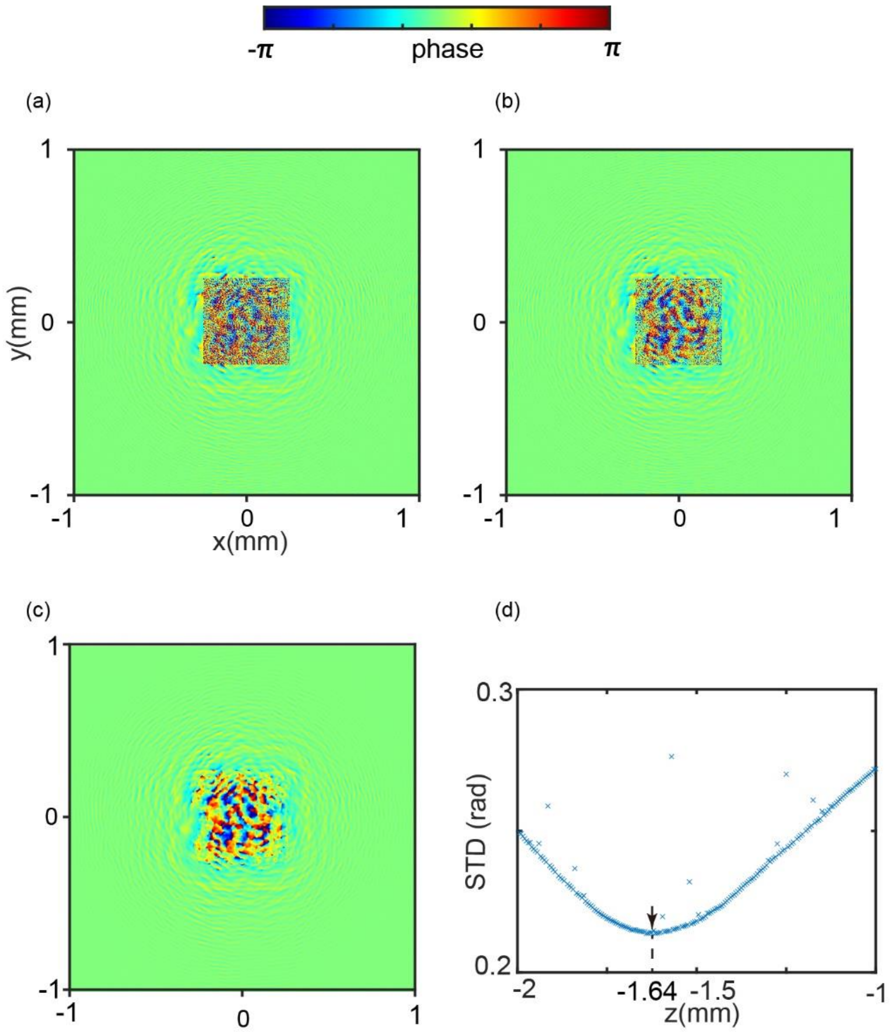

Figure S24: Simulation results of the 2 layers optical neural network. (a) The phase distribution of the bilayer network after xy-plane alignment. (b) Remove the contribution of the layer-2 from (a). (c) the low-pass filtering of (b). (d) The standard deviation between (c) and the standard distribution. The lowest point position corresponds to the deviation of the obtained layer-2 position relative to the ideal position.
